# Photographic protocol for image acquisition in craniofacial microsomia

**DOI:** 10.1186/1746-160X-7-25

**Published:** 2011-12-30

**Authors:** Carrie L Heike, Laura P Stueckle, Erik T Stuhaug, Luiz A Pimenta, Amelia F Drake, Daniela Vivaldi, Kathleen CY Sie, Craig B Birgfeld

**Affiliations:** 1 Children's Craniofacial Center, Seattle Children's Hospital, Seattle, WA, USA; 2 Department of Pediatrics, University of Washington, Seattle, WA, USA; 3 Department of Surgery, University of Washington, Seattle, WA, USA; 4 University of North Carolina's Craniofacial Center, University of North Carolina School of Dentistry, Chapel Hill, NC, USA; 5 Department of Otolaryngology-Head and Neck Surgery, University of North Carolina School of Medicine, Chapel Hill, NC, USA; 6 Department of Dental Ecology, University of North Carolina School of Dentistry, Chapel Hill, NC, USA

**Keywords:** Craniofacial microsomia, craniofacial features, digital photograph, protocol, standardize

## Abstract

Craniofacial microsomia (CFM) is a congenital condition associated with orbital, mandibular, ear, nerve, and soft tissue anomalies. We present a standardized, two-dimensional, digital photographic protocol designed to capture the common craniofacial features associated with CFM.

## Introduction

Craniofacial microsomia (CFM) is a congenital condition characterized by underdevelopment of the facial structures, typically involving the ear and mandible [1,2]. More specifically, the craniofacial malformations associated with this condition can include: **o**rbital anomalies in size and position, **m**andibular hypoplasia, **e**ar malformations (microtia), facial **n**erve palsy, and facial **s**oft tissue deficiency; all of which can be classified using the OMENS classification system [3,4]. CFM often involves one side of the face, though the condition can be bilateral. Individuals with CFM can also have dental, cardiac, renal, and cervical anomalies [1,2]. Phenotypic variability among individuals with CFM is wide, and clinicians disagree about the minimal diagnostic criteria for CFM [5-7]. For the purposes of this article, we consider CFM to include criteria listed in Table 1.

**Table 1:** Inclusion criteria for craniofacial microsomia (CFM).

**At least ONE of the following:**

• Microtia

• Anotia

• Facial asymmetry AND Preauricular tag(s)

• Facial asymmetry AND Facial tag(s)

• Facial asymmetry AND Epibulbar dermoid

• Facial asymmetry AND Macrostomia (i.e., lateral cleft)

• Preauricular tag AND epibulbar dermoid

• Preauricular tag AND macrostomia

• Facial Tag AND epibulbar dermoid

CFM has an estimated prevalence of 1:5,600 to 1:26,550 live births [8,9] and represents one of the most common conditions treated at craniofacial centers; yet, little is known about the etiology of CFM and few outcome studies are available. Multicenter studies are required to include large numbers of individuals with this condition. In order to ensure accurate phenotypic characterization of study participants recruited from multiple centers, we must develop methods to ensure high quality, standardized phenotypic data on children with CFM.

Photographs can facilitate standardized phenotypic assessment of craniofacial morphology [10]. Several photographic protocols exist for assessment of craniofacial surgical outcomes [11-13], including cleft lip repair [14-16]. To our knowledge, a published image acquisition protocol intended to capture the unique craniofacial features associated with CFM does not exist.

In this paper we present a standardized, two-dimensional, digital photographic protocol designed to capture the common craniofacial features associated with CFM.

## Methods

We developed a multicenter consortium entitled the "Facial Asymmetry Collaborative for Interdisciplinary Assessment and Learning (FACIAL)" to facilitate research on the etiology and clinical outcomes in CFM (NIDCR RC1 DE020270). Members of the craniofacial centers at four academic hospitals developed a digital photographic protocol to enable classification of the common craniofacial features coded in the phenotypic assessment tool for CFM (PAT-CFM)[17], which is based on the OMENS rating scale [3,4]. We developed an initial series of images based on prior craniofacial protocols in the literature. Team members participated in an iterative process of evaluation and modification the photographic protocol to optimize the ease of image acquisition and the quality of the resulting data. We systematically evaluated the quality of images obtained in the photo protocol in a series of 50 individuals ages 2-21 years with CFM (manuscript under review) and further refined the protocol. We developed a detailed set of instructions regarding the imaging environment and equipment, preparation of the subject, descriptions of the facial features of interest, instructions for each view, and suggestions for evaluation of the photographs. We summarized the protocol in sufficient detail to enable clinicians and researchers at multiple sites to replicate the protocol, and include a series of images and checklists to facilitate ease of use. We describe these procedures in detail below.

### The Imaging Environment and Equipment

**Figure 1:**
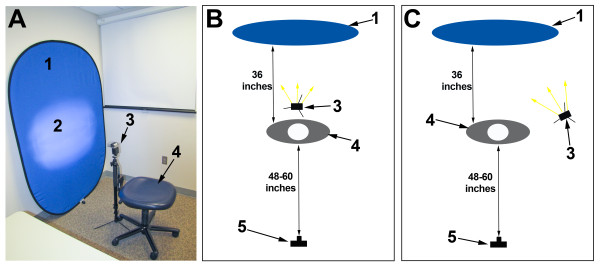
**Example of possible configurations for the image environment**. Photo of the recommended set up (A) and illustrations of a set up with the monopod flash behind the participant (B) and to the side of the participant (C). (1) Blue background, (2) flash reflection on the background, (3) monopod flash, (4) participant seat, (5) camera.

We have included several recommendations for the imaging environment, and these are illustrated in Figure 1 and summarized in Table 2.

**Table 2:** Imaging environment, equipment, and participant preparation checklist

• Select a space with sufficient room and lighting • Lighting sources include: (1) ambient, (2) camera flash, and (3) extra flash • Ensure blue background adequately covers space behind the participant
• Select seating that is appropriate for the participant
• Reposition any scalp hair that obscures the ears, face, and neck • Ask the participant to remove all jewelry from the face and ears
• Work with the participant to achieve the desired facial expression

#### Location and configuration of equipment

We recommend acquiring images in a space with minimum area dimensions of 3 meters by 1.2 meters (10 feet by 4 feet). Place the participant approximately 1 meter (3 feet) in front of the background. The camera should be 1.2 meters to 1.5 meters (4 to 5 feet) in front of the participant (Figure 1). Posting stickers, posters, or toys on the walls can cue the participant where to look during certain image acquisitions.

#### Background

We recommend using a blue background to optimize contrast for individuals with various skin tones [13]. The collapsible mat illustrated in Figure 1 works well as a portable background. The background mat should be placed against a wall so that it completely covers any structures directly behind the seated subject.

#### Lighting

Optimal images depend on adequate lighting, and studio-style lighting setups have been well-described [13]. For this portable protocol, we use the following three lighting sources: (1) ambient light (2) built-in camera flash and (3) a remote flash on a monopod (Figure 1). The ambient light in clinic or office settings is usually adequate and may include fluorescent or incandescent lights with or without natural light. The extra flashes incorporated into this protocol minimize the reliance of the ambient light, which is only important for assisting with automatic focus. The built-in camera flash should be set to "on" or "forced" so it will trigger during all image sessions. The monopod flash is used to eliminate shadows, and ideally should be placed behind the participant on a flash stand and triggered remotely. Light from the flash should not fall on the participant. There are two options for the position of the monopod flash. The optimal location is approximately 0.6 meters (2 feet) behind the participant. The flash should be positioned as low as possible and angled upward, pointing at the background just below the plane of the subject's head. The flash should remain hidden from the photographer's view by the participant's head, and should not appear in the picture. If the physical space does not allow for this, place the flash 0.6 meters (2 feet) to the left or right of the participant on the opposite side of the camera's flash (Figure 1). The flash should be arranged on the flash stand so it approximates the participant's head height. The flash should be both angled and pointed at the spot on the background directly behind the participant's head.

#### Camera settings

Though this protocol can be performed with a digital SLR camera, our protocol can be completed using a digital point and shoot minimum six megapixel camera. It is optimal to have a lens that falls between 60 mm and 105 mm. Cameras should be checked at the beginning of each photo acquisition session to ensure that the correct time and date are set, and that the settings comply with the study protocol. The camera should be in a mode that allows the operator to select the highest resolution setting and set the flash to "on" or "forced." Additional suggested settings include: Shutter sync: first curtain; Red Eye Correction: off; Red Eye Lamp: off; Wide-angle: off; Digital zoom: off. Do not use wide angle lenses as they can distort craniofacial features for a portrait. Many cameras have the default as a wide angle. If a camera has this default setting, the photographer should zoom in for each picture to avoid obtaining a wide angle photo. We recommend using the grid lines on the viewer to ensure optimal head positioning in the Frankfort horizontal plane (see "Views" section below).

#### Seating options

To maximize patient safety, we recommend placing infants and toddlers between 5 months to 3 years of age who are able to sit with minimal support in a booster chair [18]. To ensure adequate safety, we recommend that an adult stay near the child during image acquisition. For children who do not tolerate this separation from the caregiver, we recommend placing the child sideways on the caregiver's lap such that the blue mat remains in the background and the caregiver is out of the image view. Older children and adults should be placed in a chair with a low back rest to avoid interference of the seat back with the blue background. Ideally, the chair will have an adjustable seat height, and flexibility to rotate the chair to obtain the optimal positions required for image capture. Alternatively, younger children might need to stand and rotate positions for photo acquisition.

### Preparation of the Subject

We have included several recommendations for preparing participants, and these are summarized in Table 2.

#### Capturing the face and ears

The hair should be pulled back to allow for an unobstructed view of the ears. A variety of items can be used to accomplish this, including: a wig cap, a hair tie, barrettes, bobby pins, self-adhesive tape, headband, and hair rubber bands [18]. Whenever possible, subjects should remove glasses and jewelry from the face and ears, along with hearing aids [18,19]. Removal of sweatshirts with hoods, and tucking in collars and other clothing articles around the neckline facilitates adequate capture of the neck, mandible, and ear. Wiping the noses and mouth areas of infants and toddlers just prior to image capture can minimize reflection from wet surfaces that create artifacts.

#### Positioning the subject

When possible, the participant should sit on a mobile chair or an exam stool so the photographer can rotate the participant to the correct positions required for image capture. While adults and older children are frequently comfortable and safe to sit on a seat with wheels, younger children might need to stand and rotate positions for photo acquisition. Infants and young children who cannot stand should sit on a parent's lap.

#### Instructions for subjects

This protocol includes images obtained while the participant has a neutral expression, as well as during facial animation. The rationale for the expression requested for each view is described in Table 3.

**Table 3:** Common facial features affected in CFM and the image view(s) that captures these features

Feature	View
**O**rbit	View A
**M**andible	Views A-F
**E**ar	Views A- E, O, P
**N**erve	Views A, G-J
**S**oft tissue	Views A-F
**Other features**	
Occlusal cant	View K
Tongue	Views L-T
Epibulbar dermoid	Views A, M, N
Ear tags/pits	Views B-E, O, P
Facial tags/pits	Views B-E, O, P

**Figure 2:**
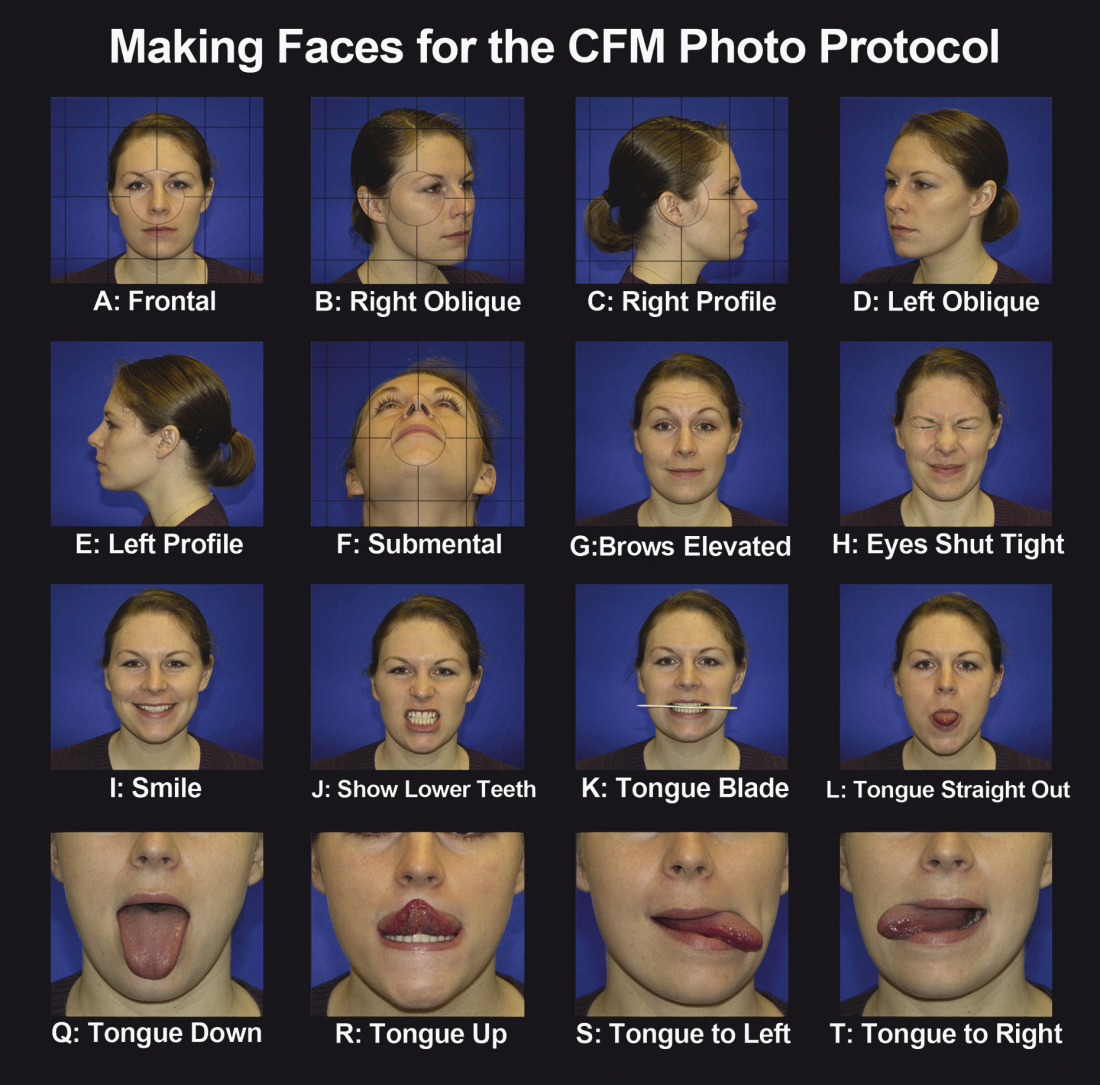
**Making Faces for the CFM Photo Protocol**. This collage illustrates optimal image acquisition for each of the views described in this protocol. This figure can be used during image acquisition to show participants examples of the requested facial expressions. The grid lines on views A-E can be used on the camera's viewer to ensure optimal head positioning in the Frankfort horizontal plane. The circle overlying images A-E represents the focal point of the image.

For neutral expressions, it is often sufficient to instruct subjects to relax their face. In addition to obvious signs of facial tension or emotional expressions, photographers should pay attention to the subject's mouth and eyes [20-22]. The subject's mouth should be closed during capture, with the lips gently pressed together. The subject's eyes should be fully open during image acquisition to allow for adequate capture of epibulbar dermoids and colobomas of the iris. A mirror may assist participants achieve the desired position and expression [23]. Older children can often follow instructions to keep neutral, relaxed face, with the mouth shut and lips gently touching [24,25]. It may also help to ask them to swallow and relax [26,27]. Younger children may require distraction devices to focus their attention in the preferred direction, and care must be taken not to elicit facial expressions (e.g., laughter or a surprised look). Such distraction devices include bubbles, toys with soft sounds and/or lights, or a children's video. We have created a template "Making Faces for the CFM Photo Protocol" (Figure 2) to show participants the types of facial expressions included in this protocol [16].

### Facial features of interest

**Figure 3:**
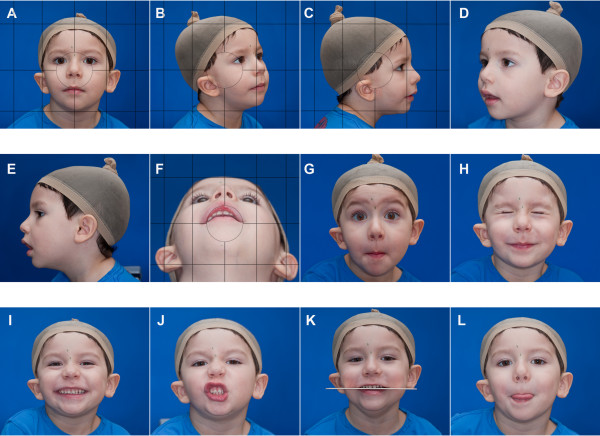
**Contact sheet used in the general evaluation in phenotypic assessment tool for CFM**. The views obtained during image acquisition can be used to create a contact sheet for quick categorization of the common craniofacial features affected in CFM. The contact sheet illustrated in this figure includes views that can be used to complete the ratings for the orbit, mandible, ear, nerve, and soft tissue in the OMENS classification system. The complete contact sheet incorporates the 16 views obtained in the protocol, in addition to 4 enlarged views of the ears and eyes as illustrated in Figure 4.

**Figure 4:**
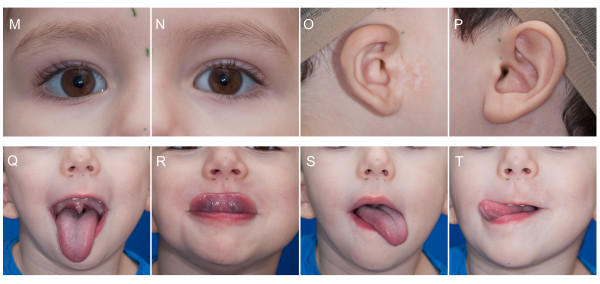
**Contact sheet used in the detailed evaluation in phenotypic assessment tool for CFM**. This page of the contact sheet is designed to allow raters to quickly assess physical features included in the "Detailed Assessment" of the phenotypic assessment tool for CFM. Enlarged views of the eyes and ears were created by enlarging views A, C, and E. Multiple views of the tongue allow for assessment of unilateral or bilateral hypoplasia.

**Figure 5:**
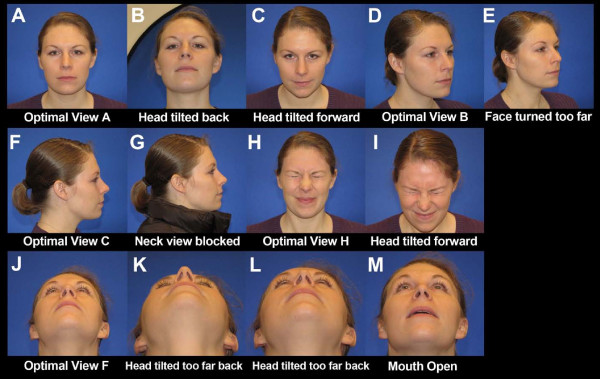
**An example of suboptimal images**. This collage provides optimal and suboptimal examples of five views. The first three images (A-C) are of View A. Image B is suboptimal for three reasons: the subject's head is tilted back, the blue background is not filling the background completely, and the photographer is angling the camera up for the picture. Image C is suboptimal for two reasons: the subject's head is tilted forward and the photographer is angling the camera down for the picture. The other views represented in this figure include View B (Images D & E), View C (Images F & G), View H (Images H & I), and View F (Images J-M). Image M is suboptimal for two reasons: the subject's mouth is open and the head is not tilted far enough back.

**Figure 6:**
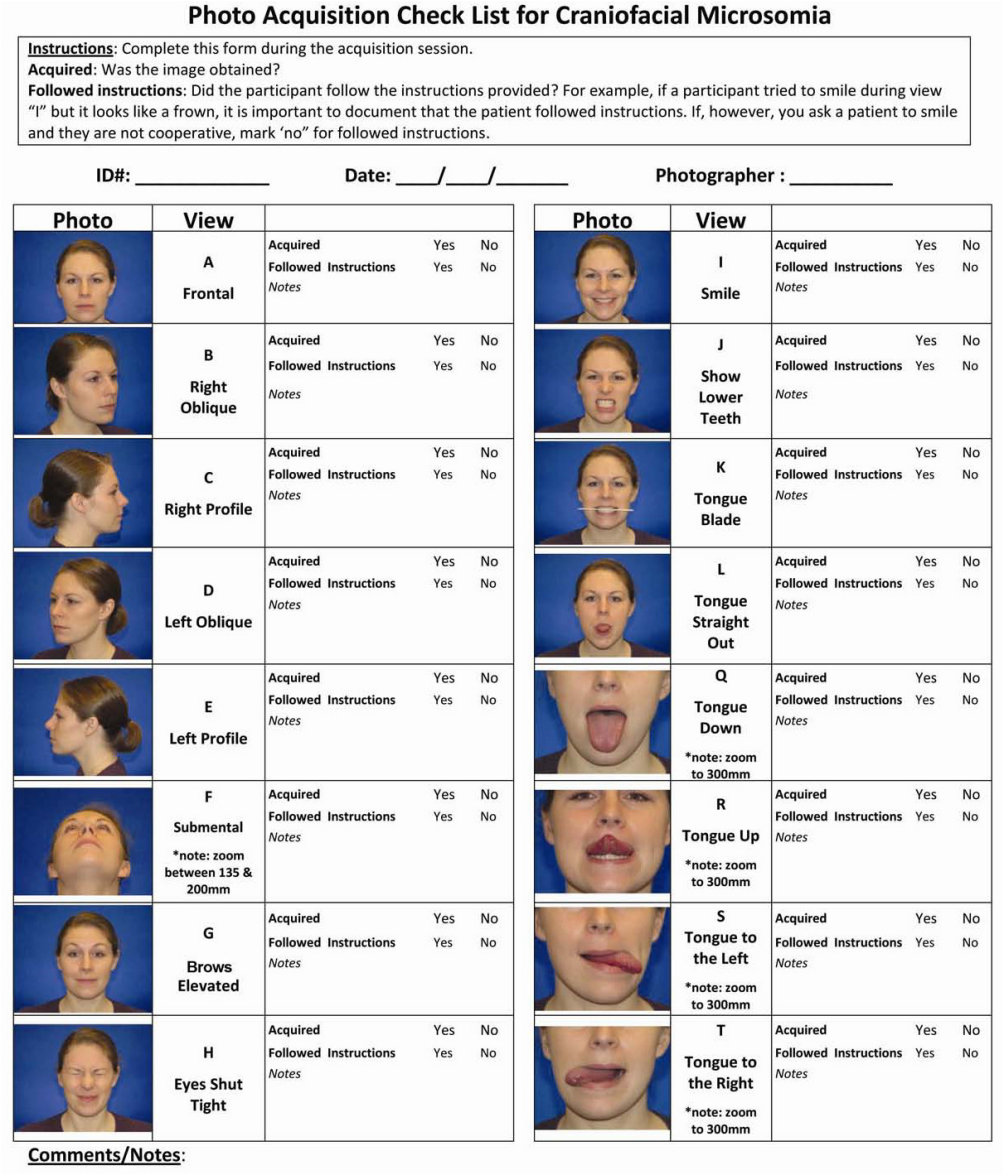
**Photo acquisition image check list**. This figure provides a check list to be used during image acquisition, along with a section to document notes about the image capture session. These data can be useful for interpretation of the reliability of the images for evaluating nerve function. For example, if the participant tried to smile during the View I, but it looks like a frown, it is important to document that the participant followed instructions. If, however, you ask the participant to smile and they are not cooperative, you would also want to document that as well.

We developed this photographic protocol to capture the facial features commonly affected in CFM and to allow for the recording of these features using the OMENS rating system. These features are briefly described below, and summarized in Tables 3 and 4, along with Figures 2, 3, 4, 5, 6.

**Table 4:** Checklist for optimal image acquisition for this CFM photo protocol

View	Name	Features	Photographer checklist	Instructions to Participant
A	Frontal	• Orbit • Mandible • Ear • Soft tissue	• Subject should always sit with his back straight • Subject should look straight forward (chin level to ground) • Subject should have a neutral expression • The plane of the face is perpendicular to the camera	• "Look at the camera with a relaxed expression." • "Close your mouth with your lips gently touching."

B	Right Oblique	• Mandible • Soft tissue	• Subject should look to his left, 45 degrees from center • Subject should have a neutral expression • Both eyes should be visible in the shot • Do not let chin drop • The tip of the nose should come to the edge of the cheek	

C	Right Profile	• Mandible • Ear • Soft tissue • Ear tags/pits • Facial tags/pits	• Subject should look 90 degrees to his left • Full profile • Center the shot directly in front of the ear • The ear canal should be visible, if present • If the ear canal is not visible from this view, take an additional photograph of the ear canal and label this "View C.2"	

D	Left Oblique	• Mandible • Soft tissue	• Subject should look to his right, 45 degrees from center • Subject should have a neutral expression • Both eyes should be visible in the shot • Do not let chin drop • The tip of the nose should in line with the edge of the cheek	

E	Left Profile	• Mandible • Ear • Soft tissue • Ear tags/pits • Facial tags/pits	• Subject should look 90 degrees to his right to obtain a full profile • Center the shot directly in front of the ear • The ear canal should be visible, if present • If the ear canal is not visible from this view, take an additional photograph of the ear canal and label this "View E.2"	

F	Sub-mental	• Mandible • Soft tissue	• Zoom half way between 135 & 200 mm • Shot should be centered on the base on the chin • Align the chin and nose • Keep face neutral with mouth closed • You should be able to see the forehead, eyes, and shape of nose • The cheekbones need to be seen • You may need to ask a parent to stand behind you and encourage the subject to look straight up for this shot • The camera will most likely need to be moved to get the shot	• "Tilt your head back and look at the ceiling."

G	Eyebrows Elevated	• Nerve	• This goal is to assess for movement of the eyebrows • NOTE: Sometimes participants are inclined to tilt their head back with this expression. Encourage them to face forward.	• "Raise your eyebrows." • "Look up with your eyes." • "Look surprised."

H	Eyes Shut Tight	• Nerve	• Subject should be centered and filling the frame • It is OK if the mouth opens and nose crinkles during this animation • NOTE: Sometimes participants are inclined to drop their chin with this expression. Encourage them to face forward.	• "Close your eyes as tightly as you can."

I	Smile	• Nerve	• Subject should be centered and filling the frame	• "Big smile."

J	Show Lower Teeth	• Lower lip depressor	• Subject should show their bottom teeth. • Showing or not showing top teeth is okay.	• "Show me your lower teeth."

K	Tongue Blade	• Occlusal cant	• Subject should show their top and bottom teeth while holding tongue blade in their mouth • We want to see how their teeth meet the tongue depressor	

L*	Tongue Straight Out	• Tongue	• Tongue should be extended as far as possible, and "pointy"	• "Stick your tongue straight out."

Q*	Tongue Down	• Tongue	• Zoom in to 300 mm and move in 1 to 1.2 meters (3 to 4 feet) from subject • Tongue should be relaxed, not be "pointy" • NOTE: Check the image to ensure it is in focus.	• "Open your mouth and hang your tongue out as far as you can."

R*	Tongue Up	• Tongue	• Move in 1 to 1.2 meters (3 to 4 feet) from subject and zoom in • NOTE: Check the image to ensure it is in focus.	• "Try to touch your tongue to your nose."

S*	Tongue to Left	• Tongue	• Move in 1 to 1.2 meters (3 to 4 feet) from subject and zoom in • NOTE: Check the image to ensure it is in focus.	• "Point your tongue to the left as far as you can."

T*	Tongue to Right	• Tongue	• Move in 1 to 1.2 meters (3 to 4 feet) from subject and zoom in • NOTE: Check the image to ensure it is in focus.	• "Point your tongue to the right as far as you can."

Views A-E, G-K should be obtained in Frankfort horizontal position Views M, N, O, and P are created by enlarging and cropping images obtained previously, thus not included in this image acquisition table

*Check to ensure that the image is in focus

#### Orbit

Malformations of the orbit in CFM commonly include small size, and inferior or superior displacement. These features are best illustrated on View A (Figure 2 and 3) and views M and N (Figure 4).

#### Mandible

Mandibular asymmetry is a hallmark of CFM and is classically attributable to malformations of the ramus. Mandibular anomalies can be difficult to fully evaluate on two-dimensional images. Our protocol incorporates multiple views of the mandible to capture mandibular hypoplasia and resulting facial asymmetry (views A-F of Figures 2 and 3, Tables 3, 4). We've also included examples of common errors in image acquisition that can lead to an inability to interpret images with regard to the mandible (Figure 5).

#### Ear

CFM is frequently associated with various grades of microtia with or without absence of the external auditory meatus. We have incorporated profile (view C and E), oblique (views B and D), and frontal view (view A) of the ear to allow for assessment of size, shape, and position. Views C and E allow for subsequent enlargement to create views O and P, respectively (Figures 3 and 4).

#### Nerve

Facial palsies can involve any or all branches of the facial nerve and may be unilateral or bilateral. We propose a series of images (views A, G-J, Figures 2 and 3) designed to capture the participant in a neutral expression, and well as animation that requires function of each branch of the facial nerve.

#### Soft tissue

Deficiency of the soft tissue is common in CFM. As described for the mandible, capturing soft tissue deficiency and the resultant facial asymmetry can be challenging using a two-dimensional imaging modality. For this reason, we've included several views of the face to allow for assessment of soft tissue asymmetry (views A-F, Figures 2 and 3).

#### Other facial features

Individuals with CFM may have additional anomalies of the face not included in the original OMENS system. For this reason, we added other common features to the CFM phenotypic assessment tool [17], including epibulbar dermoids, colobomas of the upper lid, ear tags, facial tags, preauricular pits, facial pits, macrostomia, clefts of the lip, occlusal cant, and tongue hypoplasia. The images captured in this photographic protocol (described below) can be used to assess for these features. View A can be enlarged to create views M and N for evaluation of the eyes (Figure 4). Similarly, enlargement of views C and E allows for detailed assessment of the ears in Views O and P (Figure 4).

### Views

We identified 16 image views that collectively capture the facial features described above. Views (A-E, G-L) should taken with the participant in the Frankfort horizontal plane, which is achieved by ensuring the lower margins of the orbits are on the same level as the upper margins of the ear canals. This can be challenging for participants with microtia and/or orbital displacement. The goal is to obtain a full frontal view, in which the face is perpendicular to the camera. Participants should be optimally positioned on the horizontal and vertical axes to eliminate rotation from the midline [11]. The grid lines on views A-C, F can be used on the camera's viewer to ensure optimal head positioning in this plane [11].

Oblique views should be obtained in the Frankfort horizontal position, and the participant should face approximately 45 degrees away from the camera [11]. The photographer should attempt to align the profile of the face with the opposite cheek, as illustrated in Views B and D. Profile views should be obtained with the participant facing at an angle 90 degrees away from the photographer [11], as illustrated in Views C and E.

As previously described, views M, N, O, and P (Figure 4) can be generated by enlarging and cropping views (A, C, and E) from Figure 3. We illustrate the views obtained during image acquisition in Figures 2 and 3, and demonstrate the full set of 20 images (including 4 created during image processing) used in the contact sheet in Figures 3 and 4. We have illustrated optimal images, as well as common factors that contribute to suboptimal images (Figure 5). We have also included an image acquisition checklist to be used during image acquisition sessions for interpretation of data during image analysis (Figure 6).

### Evaluation of the photographs at the time of image capture

We encourage photographers to preview images at the time of image acquisition to minimize the possibility of missing data during image acquisition. Reviewing images for key features (Table 4) at the time of image capture while the participant is present allows the opportunity to acquire additional captures at that time [24].

It may not be feasible to review all images at the time of image acquisition, particularly when photographing small children. In this case, photographers can acquire multiple images for each view to maximize the likelihood of obtaining adequate data coverage, and process the images later for subsequent evaluation.

### Photographic interpretation

We have developed an image protocol designed to capture the common craniofacial anomalies associated with CFM. These images can be assessed individually, or they can be combined to create a composite sheet for each individual. In the PAT-CFM tool, we used the views described in this protocol to create a composite sheet that would enable efficient classification of the features included in the modified pictorial OMENS-plus classification system [17]. We used a commercial software program to automate the creation of contact sheets for study participants and many programs can create similar results. Examples of the views we've included in the contact sheet are illustrated in (Figure 3).

## Conclusion

Photographic records can serve as a powerful resource to capture and quantify craniofacial morphology. Acquiring reliable, high-resolution, and high-quality facial images requires standardized methods to optimize image acquisition.

We developed this protocol with the multidisciplinary, multicenter FACIAL team and used an iterative process of testing to develop a stepwise procedure to allow for a person with limited photographic training to be able to acquire high quality, standardized images that capture the most common malformations associated with CFM. We designed a protocol that could be incorporated into low-resource settings using a digital camera with reasonable resolution. We also aimed to create a protocol that was not overly burdensome for the study participants and/or patients, and allows for subsequent classification of images outside of the clinical or research encounter.

The data collected via this protocol allows for future assessment of images using the OMENS classification system, along with other classification systems. We also anticipate that the standardized protocol may enable clinicians and researchers to document and assess changes over time, either with growth and/or intervention. Though we anticipate that reliability of ratings on the photos compared to ratings by in-person physical examination will be high for many of the malformations assessed in this protocol, future reliability studies are needed.

Not all participants will be able to comply with the instructions, particularly infants and young children, and this will result in missing data. This is likely also true for some features on direct physical examination, such as facial nerve exams in neonates. Future reliability studies that include large numbers of participants of all ages will be helpful to further assess the reliability of the protocol and completeness of data collection across age-groups.

Our assessment of facial nerve function is relatively simple. It is a first step in indirect assessment of facial nerve palsies in CFM and can be complemented with a video or clinical examination. Though we anticipate the reliability of the entire protocol will be high for older, cooperative participants for the anatomical components of the face, it will be important to formally evaluate the reliability of this photographic protocol, particularly for the nerve function.

As with any study protocol, it is up to the reader to determine the applicability of the aforementioned techniques to their specific research or clinical question. Though this protocol may be helpful to document the primary malformations associated with CFM and establish a pre-surgical baseline (i.e. grade 2 vs 3 microtia), the protocol needs to be further evaluated to determine its utility in assessment of treatment outcomes.

## Consent

As this participant is under the age of 18, written informed consent was obtained from the participant's parent for publication of this methodology and accompanying images. A copy of the written consent is available for review by the Editor-in-Chief of this journal.

## List of abbreviations

CFM: craniofacial microsomia; FACIAL: Facial Asymmetry Collaborative for Interdisciplinary Assessment and Learning; OMENS: classification system for **o**rbital anomalies in size and position, **m**andibular hypoplasia, **e**ar malformations (microtia), facial **n**erve palsy, and facial **s**oft tissue deficiency.

## Competing interests

The authors declare that they have no competing interests. The authors of this work do not have any financial disclosures or commercial associations with any imaging device/company that might pose or create a conflict of interest with the information in this manuscript.

## Authors' contributions

CH, LS, and CB conceptualized the paper. All authors contributed to content of the manuscript and development of the protocol. CH, LS, ES, DV, AD, LP, KS, and CB drafted and edited the manuscript. All authors have read and approved the final manuscript.

## Authors' information

CH, LS, KS, CB and ES are affiliated with the Children's Craniofacial Center at Seattle Children's Hospital, Seattle, WA. CH is an Assistant Professor in the Department of Pediatrics at the University of Washington, Seattle, WA. CB is an Assistant Professor in the Department of Surgery, Division of Plastic Surgery at the University of Washington. KS is a Professor in the Department of Surgery, Division of Otolaryngology at the University of Washington. LP, AD, and DV are affiliated with the University of North Carolina's Craniofacial Center at the University of North Carolina School of Dentistry, Chapel Hill, NC. AD is a Professor of Otolaryngology, Head and Neck Surgery, and Director of University of North Carolina Craniofacial Center. LP is the Dental Director at the University of North Carolina Craniofacial Center, School of Dentistry and University of North Carolina at Chapel Hill. DV is an Adjunct Professor in the Department of Dental Ecology, School of Dentistry at the University of North Carolina at Chapel Hill.
